# tRF-5028c disrupts trophoblast function in recurrent spontaneous abortion by inhibiting CRKL-mediated Rap1 signaling pathway

**DOI:** 10.1186/s11658-025-00706-w

**Published:** 2025-03-05

**Authors:** Jialyu Huang, Jiawei Wang, Shuang Wang, Xiangpeng Xiong, Ruiyin Jiang, Chaoyi Xiong, Lu Wang, Lingling Huang, Yan Zhao, Zheng Fang, Xiaoyan Ai, Jiaying Lin

**Affiliations:** 1 Center for Reproductive Medicine, Jiangxi Key Laboratory of Reproductive Health, Jiangxi Maternal and Child Health Hospital, Jiangxi Branch of National Clinical Research Center for Obstetrics and Gynecology, Nanchang Medical College, Nanchang, China; 2 Reproductive and Genetic Hospital, The First Affiliated Hospital of USTC, Division of Life Sciences and Medicine, University of Science and Technology of China, Hefei, China; 3 Department of Gynecology, Jiangxi Maternal and Child Health Hospital, Nanchang Medical College, 318 Bayi Avenue, Nanchang, 330006 China; 4 Department of Clinical Medicine, School of Queen Mary, Nanchang University, Nanchang, China; 5 Department of Pathology, Jiangxi Maternal and Child Health Hospital, Nanchang Medical College, Nanchang, China; 6 Center for Reproductive Medicine, Department of Obstetrics and Gynecology, Tangdu Hospital, Air Force Medical University, 569 Xinsi Road, Xi’an 710038, China; 7 Department of Assisted Reproduction, Shanghai Ninth People’s Hospital, Shanghai Jiao Tong University School of Medicine, 639 Zhizaoju Road, Shanghai, 200011 China

**Keywords:** Recurrent spontaneous abortion, Trophoblast, tRF-5028c, CRKL, Rap1

## Abstract

**Background:**

Recurrent spontaneous abortion (RSA) affects approximately 1–5% of childbearing women and poses a significant threat to global reproductive health. Transfer RNA-derived small RNAs (tsRNAs) are a novel class of noncoding RNAs implicated in various human diseases. However, the role and mechanism of tsRNAs in regulating trophoblast function during RSA development remain unknown.

**Methods:**

High-throughput sequencing was performed to analyze the differential tsRNAs in the villous tissues of patients with RSA and controls. CCK-8, transwell assay, and flow cytometry were performed to detect the effects of tRF-5028c on proliferation, migration, invasion, and apoptosis of human extravillous trophoblast cell line HTR-8/SVneo. The target genes of tRF-5028c were predicted via bioinformatic analysis and verified by dual luciferase reporter gene assay. Moreover, pregnant mice were injected with tRF-5028c mimics to confirm the findings in vivo.

**Results:**

A total of 1907 tsRNAs were detected, of which 298 were differentially expressed in the villous tissues. tRF-5028c was significantly upregulated in the RSA group compared with control. Functionally, tRF-5028c overexpression inhibited HTR-8/SVneo cell proliferation, migration, and invasion and promoted apoptosis, whereas tRF-5028c knockdown showed opposite effects. Mechanically, tRF-5028c suppressed CRKL expression by directly binding to its 3′-untranslated region, thus inactivating the downstream C3G/Rap1 signaling pathway. Finally, tRF-5028c mimics injection increased embryo absorption rate in mice.

**Conclusions:**

tRF-5028c upregulation impaired trophoblast function to facilitate RSA development by directly targeting CRKL-mediated Rap1 pathway. The findings provide the first evidence of tsRNA dysregulation in RSA pathogenesis and lay a foundation for potential targeted therapies.

**Supplementary Information:**

The online version contains supplementary material available at 10.1186/s11658-025-00706-w.

## Introduction

Recurrent spontaneous abortion (RSA), defined as the occurrence of two or more spontaneous abortions before 20 weeks of gestation, is observed in 1–5% of childbearing women and poses a significant threat to global reproductive health [1]. The etiologies of RSA have been commonly attributed to chromosomal abnormalities, uterine anatomical defects, as well as infection, endocrine, and immune factors [2]. However, around 50% of RSA remains unexplained with no known causes, implying the need for a deeper understanding of its pathogenesis.

Trophoblasts are a heterologous population of cells derived from the outer layer of embryos, including cytotrophoblasts (CTB), syncytiotrophoblasts (STB), and extravillous trophoblasts (EVT) [3]. These cells play a crucial role in embryo implantation and early pregnancy [4]. Notably, insufficient EVT migration and invasion can disrupt the maternal–fetal interface connection, leading to inadequate placentation and consequently increased risks of RSA, preeclampsia, and fetal growth restriction [5]. Therefore, investigating the molecular mechanisms underlying trophoblast dysfunction is essential for the development of novel treatment strategies for RSA.

Transfer RNA-derived small RNAs (tsRNAs) are unique short noncoding RNAs originated from precursor or mature tRNAs [6]. Based on their length and cleavage location, tsRNAs can be classified into two primary types: tRNA halves (tiRNAs) and tRNA-derived fragments (tRFs) [7]. The tRFs are further subdivided into tRF-1-3 and tRF-5 depending on the specific tRNA cleavage sites [8]. Emerging studies have highlighted the involvement of tsRNAs in a variety of biological processes and human diseases, especially in cancer [9–13]. For instance, tRF-20-S998LO9D, which is low expressed in endometrial carcinoma, was shown to suppress cell proliferation, migration, and invasion [14]. Conversely, upregulation of tsRNA-26576 promoted the tumorigenesis of breast cancer [15]. Nonetheless, the role of tsRNAs in RSA remains unclear and warrants further exploration.

Several potential pathways have been proposed for tsRNAs in gene expression regulation, one of which is an RNA interference-like mechanism. Similar to microRNAs, tsRNAs can bind to Argonaute (AGO) proteins and guide the RNA-induced silencing complex (RISC) to target mRNAs based on sequence complementarity, thus leading to repression of its translation or degradation [10]. In this study, our sequencing revealed an overexpression of tRF-5028c (tRF-1:31-Glu-TTC-2) in the villous tissue of patients with RSA, whose target genes were functionally enriched in diverse signaling pathways including Ras‐associated protein‐1 (Rap1) pathway. Rap1 is a small GTP‐binding protein activated by various extracellular stimuli, such as growth factors and cytokines [16]. Previous investigations have evidenced that Rap1 is expressed in the placenta and its knockdown could impede trophoblast migration and invasion [17]. Subsequently, though intersecting the target genes of tRF-5028c and Rap1 pathway genes, v-crk sarcoma virus CT10 oncogene homolog-like (CRKL) was identified as a potential candidate. CRKL, a family member of the Crk adaptor proteins, encodes SH2 and SH3 domains that facilitate the assembly of multiprotein complexes by binding to various adaptor molecules [18]. Importantly, CRKL is required for gene transcription and activation of Rap1 [19]. However, the interplays between tRF-5028c, CRKL and Rap1 in modulating trophoblast biology are yet to be determined.

Collectively, we hypothesize that tRF-5028c inhibits the Rap1 pathway by targeting CRKL expression, thereby suppressing trophoblast migration and invasion and ultimately exacerbating RSA progression. Our findings provide the first evidence of tsRNA dysregulation in RSA, which further contributes to the understanding of its pathogenic mechanisms and lays a foundation for developing targeted therapies.

## Materials and methods

### Clinical sample collection

Between March 2022 and April 2023, 15 patients with unexplained RSA and 15 patients with normal pregnancy terminations for nonmedical reasons were enrolled from the Department of Gynecology at Jiangxi Maternal and Child Health Hospital affiliated to Nanchang Medical College. All participants were nonsmokers aged under 38 years old and had normal body mass index (18.5–24 kg/m^2^). Patients in the control group had no adverse pregnancy history, such as spontaneous abortion, preeclampsia, or preterm birth. RSA was defined as two or more previous spontaneous abortions before 20 weeks of gestation [1], and patients with the following conditions were excluded: (1) parental or embryonic karyotype abnormalities by chromosomal analysis, (2) uterine malformations or cervical incompetence by pelvic examination, three-dimensional (3D) ultrasound, or hysteroscopy; (3) genital tract infection by vaginal swab pathogen test; (4) endocrine or metabolic dysfunction (e.g., hyperprolactinemia) by comprehensive hormonal assessment; (5) history of autoimmune diseases (e.g., systemic lupus erythematosus) by immunological antibody screening; and (6) other known causes of miscarriage. Placental villous tissues were collected immediately after curettage at 6–10 weeks of gestation. After washing with PBS, all samples were separated for two parts and fixed in 4% paraformaldehyde or stored in liquid nitrogen. Characteristics of the control and patients with RSA are detailed in Supplementary Table S1. The study was approved by the Reproductive Medicine Ethics Committee of Jiangxi Maternal and Child Health Hospital (no. 2022-09), and written informed consent was obtained from each patient.

### tsRNA sequencing

tsRNA sequencing was performed on villous tissues of three RSA cases and three controls. In brief, RNA samples were preprocessed by 3′-aminoacyl deacylation to 3′-OH, 3′-cP removal to 3′-OH, 5′-OH phosphorylation to 5′-P, m1A and m3C demethylation, and then subjected to library preparation using the NEBNext^®^ Multiplex Small RNA Library Prep kit (New England BioLabs, MA, USA; E7330S). Subsequently, the prepared libraries were sequenced using the Illumina NextSeq 500 system (Illumina, CA, USA). The expression levels of tsRNAs in the high-throughput sequencing profiles were quantified as counts per million mapped reads (CPM). Based on fold change (cutoff 1.5) and *P*-value (cutoff 0.05), differentially expressed tsRNAs between groups were identified using the R package edgeR, which were further visualized in hierarchical clustering heatmap and volcano plot with the ggplot2 and heatmap2 packages. Potential tsRNA target genes were predicted via miRanda and TargetScan, and a tsRNA-mRNA network was constructed in Cytoscape. Functional enrichment of the putative tsRNA targets was also performed using Gene Ontology (GO) and Kyoto Encyclopedia of Genes and Genomes (KEGG) analyses, with significance set at *P* < 0.05.

### Immunohistochemistry (IHC)

Following deparaffinization and antigen retrieval (Dako, CA, USA; S1699), the tissue slices were blocked and treated overnight with antibodies against CRKL (Abcam, Cambridge, UK, 1:100, ab32018) or IgG (Abcam, 1:100, ab6759). The sections were then treated for 1 h with the secondary antibody (Abcam, 1:500, ab150077). Subsequently, they were reacted with DAB solution, stained with hematoxylin, air-dried, and sealed with neutral gum. The photos were captured with an Olympus microscope (Tokyo, Japan).

### Immunofluorescence staining

The villous tissues were fixed using 4% paraformaldehyde, rinsed with PBS, and permeabilized with 0.1% Triton-X100. Subsequently, the tissue slices were incubated overnight at 4 ℃ with primary antibodies against CRKL (Abcam, 1:50, ab32018), HLA-G (Abcam, 1:100, ab283260) and CK7 (Abcam, 1:100, ab181598). Sections were then incubated with secondary antibodies conjugated with Alexa Fluor^®^ 488 (Abcam, 1:500, ab150113) or 594 (Abcam, 1:500, ab150116) at 25 ℃ for 1 h. Finally, the slices were counterstained with 4′,6-diamidino-2-phenylindole (DAPI) (ThermoFisher Scientific, MA, USA; D1306) and visualized under a Zeiss LSM 880 confocal microscope (Oberkochen, Germany).

### Cell culture and transfection

An immortalized EVT cell line, HTR-8/SVneo (ATCC, VA, USA), was cultured in DMEM (Gibco, MD, USA; 11965092) mixed with 10% FBS (Gibco; A5669701) in a 37 °C humidified chamber containing 5% CO_2_. The inhibitor/mimic of tRF-5028c, short hairpin RNA of C3G (sh-C3G), overexpression plasmid of CRKL (oe-CRKL) and their negative controls (NCs) were provided by GenePharma (Shanghai, China). According to the manufacturer’s instructions, the inhibitor/mimic, shRNA and/or plasmid were transfected into cells using Lipofectamine 3000 (Invitrogen, CA, USA; L3000150). The sequences of inhibitor/mimic of tRF-5028c and NC were listed as follows: tRF-5028c inhibitor: 5′-CCAGGAAUCCUAACCGCUAGACCAUGUGGGA-3′, inhibitor NC: 5′-AAUAGACCGGUGCGGCUAAACAGCAUCGCUC-3′; tRF-5028c mimic forward: 5′-UCCCACAUGGUCUAGCGGUUAGGAUUCCUGG-3′, mimic NC forward: 5′-GAGCGAUGCUGUUUAGCCGCACCGGUCUAUU-3′; tRF-5028c mimic reverse: 5′-AGGAAUCCUAACCGCUAGACCAUGUGGGAUU-3′, mimic NC reverse: 5′-UAGACCGGUGCGGCUAAACAGCAUCGCUCUU-3′.

### Fluorescence in situ hybridization (FISH)

A commercial FISH Kit (RiboBio, Guangzhou, China; C10910) was used for this assay. Sequentially, the cells were washed with cold PBS, fixed with 4% paraformaldehyde for 13–15 min, and permeabilized with 0.5% Triton X-100 containing PBS for another 4–5 min. Cells were then incubated overnight with fluorescence-labeled probes specific for tRF-5028c at 37 ℃. After washing, cells were placed on slides with DAPI-containing mounting media (ThermoFisher Scientific, MA, USA; D1306) followed by confocal imaging (Zeiss LSM 900, Germany).

### Cell counting kit-8 (CCK-8) assay

Cells were cultured in 96-well plates (2 × 10^4^ cells/well) for different time periods (0, 24, 48, and 72 h). Then, 10 nmol/L CCK-8 solution (10 μL, Sangon, Shanghai, China; E606335) was added and incubated at 37 ℃ for 3 h. Absorbance was examined at 450 nm.

### Transwell migration and invasion assay

Transfected cells were collected and resuspended in serum-free medium (1 × 10^6^ cells/mL). Cell suspension (100 μL) was then added in the bottom chamber, while medium containing 10% FBS (700 μL) was placed in the lower chamber. After 12 h, cells on the top chamber were removed, while cells on the bottom chamber were fixed and stained with 0.5% crystal violet. An Olympus microscope was used to image cells. When executing the transwell invasion assay, the top chamber was additionally precoated with 1:8 Matrigel (Corning, NY, USA; 356,234).

### Cell apoptosis assay

Cells were resuspended in the binding buffer (Beyotime, Shanghai, China; C1062S) and incubated with 5 µL Annexin V-FITC and 5 µL propidium (PI) for 15 min in the dark. The samples were then subjected to flow cytometry (BD, NJ, USA) analysis. Annexin V-FITC ( +) and PI (–) indicated apoptotic cells.

### Dual luciferase reporter gene assay

The 3′-UTR of CRKL was amplified by PCR and subjected to site-directed mutagenesis. Firstly, we integrated the miRanda and TargetScan algorithms and performed computational analysis to identify potential binding sites between tRF-5028c and CRKL. Based on the principle of complementary base pairing, we mutated T to A and G to C, thereby designing corresponding primers to generate the mutated sequences (…AT…ACCC…). Wild-type (WT) and mutant (MUT) reporter plasmids of CRKL 3′-UTR were then cloned into the pGL3 vector (Promega, WI, USA; E1751). Cells were transfected with the above vectors and tRF-5028c mimic or mimic NC, and the luciferase activity was subsequently tested. The Renilla luciferase functioned as the internal control for normalization.

### Pull-down assay

Pull-down assay for GTP-bound RAP1 was performed using the RAP1 activation assay kit (Millipore, MA, USA; 17-321). All operations were strictly carried out in accordance with the manufacturer’s instructions. In brief, cells were first incubated with lysis buffer for 30 min, and the cell lysates were treated with 200 μM 8-pCPT for 10 min to activate Rap1. The mixtures were subsequently incubated with a GST-Rap-binding domain and then pulled down through anti-GST antibody (Abcam, 1:20, ab111947). The pulled-down complexes were analyzed by immunoblotting with anti-RAP1 antibody (Abcam, 1:500, ab14404).

### Animal experiments

Mature Kunming strain mice (6–8 weeks, 25–30 g) were purchased from SJA LABORATORY (Hunan, China) and maintained in a controlled environment with free access to food and water. The animal experiments were performed as previously described [20]. Female mice were mated with male mice overnight in a 2:1 ratio. The vaginal plug was examined for pregnancy on the second morning recorded as GD 0.5. Pregnant mice were randomly injected with 10 nmol (dissolved in 200 μL PBS) of tRF-5028c mimic (*n* = 6) or mimic NC (*n* = 6) through the tail vein from GD5.5 every 3 days. On GD13.5, mice were euthanized to assess the embryo absorption rate and the placental tissues were collected for further analysis. All animal experiments were reviewed and approved by the Animal Ethics Committee of Nanchang Medical College.

### Hematoxylin and eosin (HE) staining

Formalin-fixed paraffin-embedded mouse placentas were sectioned at 4 μm thickness. The sections were deparaffinized in xylene and rehydrated through a graded ethanol series. HE staining was performed sequentially with Mayer’s hematoxylin (Sigma-Aldrich; 51275) and Eosin Y solution (Sigma Aldrich; 318906). Subsequently, the slides were dehydrated through increasing concentrations of ethanol, cleared in xylene, and mounted with a coverslip using resin-based medium. Stained sections were examined under a light microscope (Olympus).

### Quantitative real-time polymerase chain reaction (qRT-PCR)

Cells were lysed in TRIzol (ThermoFisher Scientific, MA, USA; 15596018CN), and the NanoDrop 2000 was applied for RNA concentration and quality quantification. Total RNA (1 μg) was reversely transcribed into cDNA with the Advantage^®^ RT-for-PCR Kit (Takara, Tokyo, Japan; 639505). SYBR (ThermoFisher Scientific; A57155) was applied to test the expression of mRNA. The expression of tsRNA was detected using the Bulge-Loop miRNA qRT-PCR Stater Kit (RiboBio; C10211-1) with specific stem-loop RT primers. GAPDH and U6 were employed as the reference genes for mRNA and tsRNA quantification, respectively. The data were analyzed using the 2^−ΔΔCT^ method. The primers used in the study are listed at Supplementary Table S2.

### Western blot

A BCA kit from Beyotime (Shanghai, China; P0009) was used to measure the protein concentration after extraction with RIPA buffer (Beyotime; P0013B). SDS-PAGE (10%) was used to separate the protein (20 μg), which was then transferred to PVDF membranes (Millipore; ISEQ00010). The membranes were blocked and incubated overnight with antibodies against C3G (Abcam, 1:1000, ab251683), CRKL (Abcam, 1:1000, ab32018) and GAPDH (Abcam, 1:5000, ab8245), followed by hybridization with the secondary antibody (Abcam, 1:5000, ab7090) for 60 min. ECL (Beyotime; P0018S) was used to visualize the protein bands, and the grayscale was evaluated by ImageJ.

### Statistical analysis

All statistical analyses were performed using GraphPad Prism 9.0 (GraphPad Inc., CA, USA). Data are presented as mean ± standard deviation (SD). The differences between the two groups were investigated using Student’s *t*-tests. One-way ANOVA with Tukey’s post hoc test was performed to compare differences among multiple groups. *P* values less than 0.05 were regarded as statistically significant.

## Results

### tRF-5028c was highly expressed in the villous tissues of patients with RSA

**Fig. 1:**
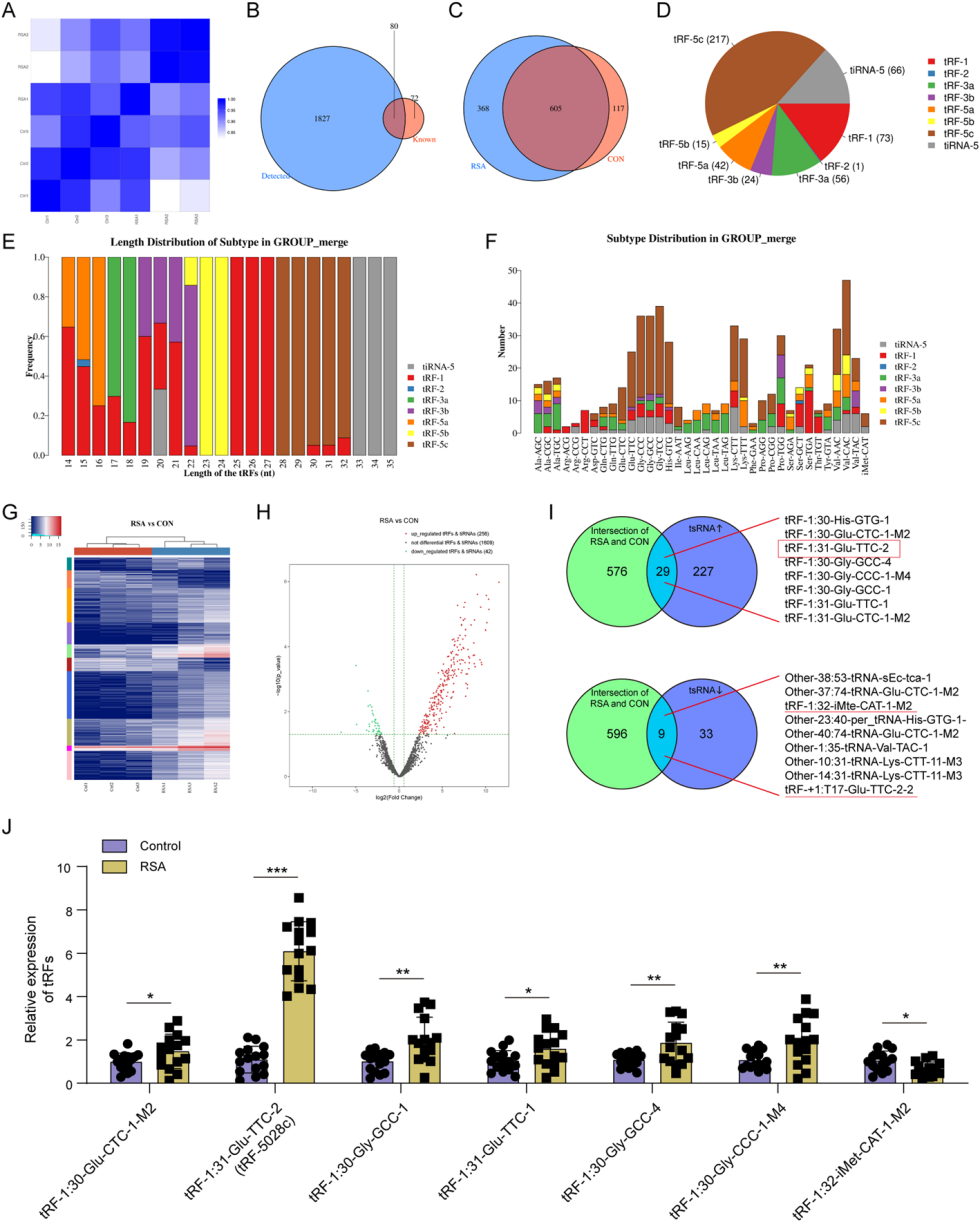
tRF-5028c was highly expressed in the villous tissues of patients with RSA. **A** Pearson correlation analysis of tsRNA sequencing data in villous tissues of three RSA cases and three controls. The correlation coefficients are greater than 0.8 among all samples. **B** Venn diagram showing the number of detected tsRNAs and known tsRNAs in tRFdb. **C** Venn diagram showing the number of commonly expressed tsRNAs in RSA and control groups. **D** Pie diagram showing the distribution of different tsRNA subtypes. **E** Length distribution analysis of tsRNA subtypes. **F** Number of tsRNA subtypes against transfer RNA isodecoders. **G**-**H** Hierarchical clustering heatmap and volcano plot of differentially expressed tsRNAs in RSA and control groups. The criteria for differential expression are based on both fold change (cutoff 1.5) and *P*-value (cutoff 0.05). **I** The 605 commonly expressed tsRNAs in both groups were intersected with the 256 significantly upregulated tsRNAs (upper panel) and the 42 significantly downregulated tsRNAs (lower panel) in RSA, respectively. **J** Expression validation of seven tsRNAs by qRT-PCR in the villous tissues of 15 patients with RSA and 15 controls. Data presented as mean ± SD. *p < 0.05, **p < 0.01, ***p < 0.001

Using high-throughput sequencing, we first analyzed tRF & tiRNA expression patterns in the villous samples of patients with RSA and controls. All six samples exhibited correlation coefficients greater than 0.8 (range 0.82 to 0.99), indicating a high level of similarity and data reliability for further investigation (Fig. 1A). A total of 1907 tsRNAs were detected, of which 80 were matched to the tRFdb (http://genome.bioch.virginia.edu/trfdb/) and 1827 were novelly identified (Fig. 1B). Among the 1090 tsRNAs with CPM ≥ 20, 605 were commonly expressed in both groups (Fig. 1C). tRF-5, especially tRF-5c, constituted the most abundant type of tsRNAs (Fig. 1D). The length distribution analysis showed that tiRNA-5 were mostly at 33–35 nt, tRF-1 at 25–27 nt, tRF-2 at 15 nt, tRF-3a at 17–18 nt, tRF-3b at 19–22 nt, tRF-5a at 14–16 nt, tRF-5b at 23–24 nt, and tRF-5c at 28–32 nt (Fig. 1E). The number of subtypes against tRNA isodecoders was also evaluated, which implied a nonrandom origin, such as tRF-3a in favor of Ala-, Gln-, Pro-, and Leu-tRNA and tRF-1 in favor of Ser-, Thr-, and especially Arg-tRNA (Fig. 1F).

As shown in Fig. 1G, H, 256 significantly upregulated and 42 downregulated tsRNAs were identified in the RSA group compared with control. When cross-referencing the upregulated tsRNAs with the 605 commonly expressed tsRNAs in both groups, 29 were found to be upregulated (upper panel of Fig. 1I). Similarly, intersecting the common tsRNAs with the downregulated ones revealed 9 tsRNAs exhibiting downregulation in RSA (lower panel of Fig. 1I). After excluding tsRNAs classified as “others,” we first obtained 10 tsRNAs (8 upregulated and 2 downregulated) as potential candidates. Given the predominant abundance of tRF-5c in villous tissue, tRF± 1:T17-Glu-TTC-2-2 in the tRF-1 group was then excluded. To avoid potential sequencing outliers, we additionally excluded tRF-1:30-His-GTG-1 with the highest upregulation (9.26-fold) and tRF-1:31-Glu-CTC-1-M2 with the lowest upregulation (5.16-fold). Subsequently, using an expanded sample size of patients with RSA (*n* = 15) and controls (*n* = 15), the differential expressions of the remaining 7 tsRNAs were validated by qRT-PCR, including tRF-1:30-Glu-CTC-1-M2, tRF-5028c, tRF-1:30-Gly-GCC-1, tRF-1:31-Glu-TTC-1, tRF-1:30-Gly-GCC-4, tRF-1:30-Gly-CCC-1-M4, and tRF-1:32-iMet-CAT-1-M2 (Fig. 1J). The results were consistent with the sequencing data, and tRF-5028c was found to be the most significantly overexpressed in RSA and was thus chosen for the following study.

### tRF-5028c regulated the function of trophoblast cells

**Fig. 2:**
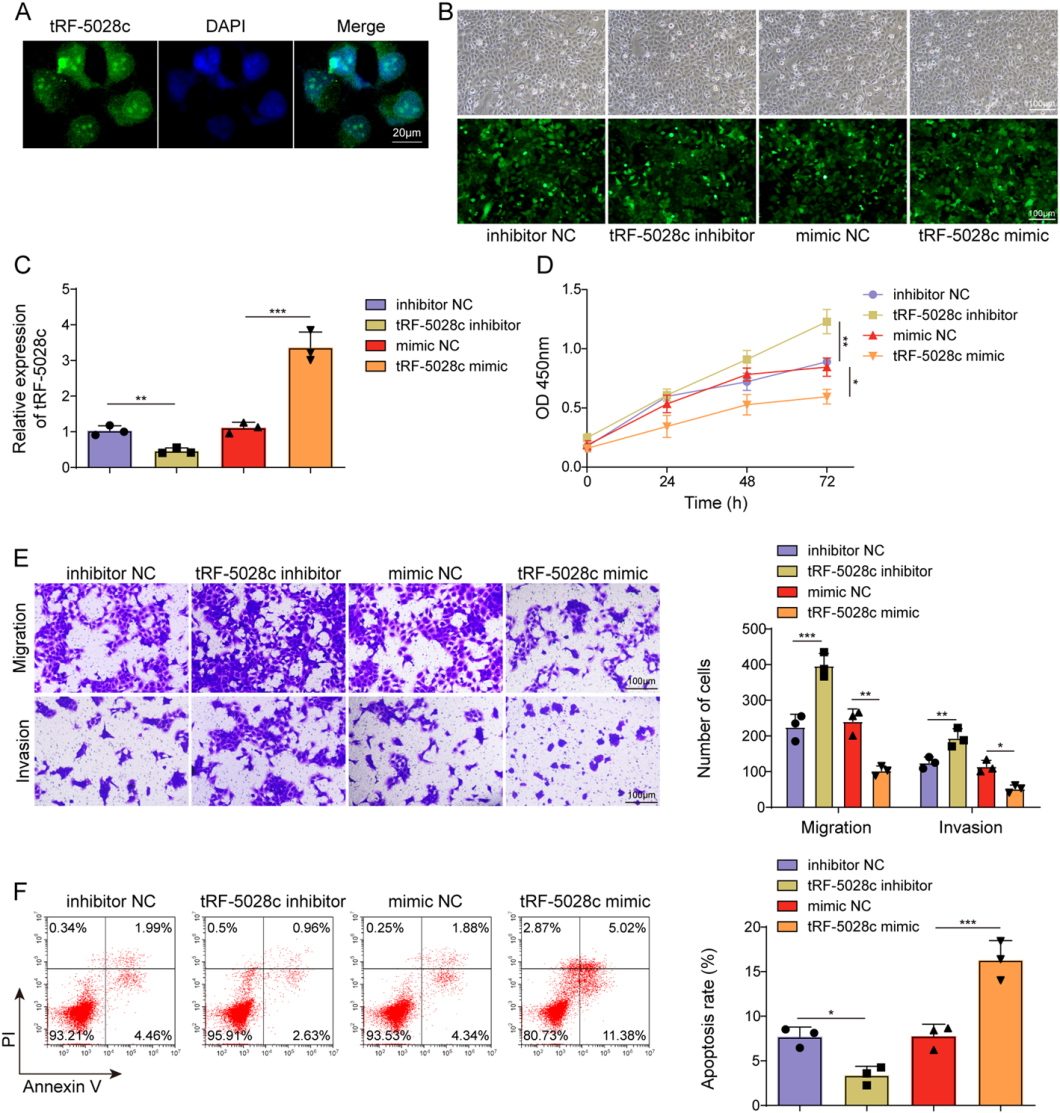
tRF-5028c promoted trophoblast proliferation, migration, and invasion and inhibited apoptosis. **A** tRF-5028c location in HTR-8/SVneo cells was analyzed by FISH assay. HTR-8/SVneo cells were transfected with tRF-5028c inhibitor/mimic or inhibitor/mimic NC. **B**, **C** The transfection efficiencies were detected by fluorescence microscopy and qRT-PCR after 24 h. **D** CCK-8 assay was performed to evaluate transfected HTR-8/SVneo cell proliferation at 0, 24, 48, and 72 h. **E** Transwell assay was performed to evaluate transfected HTR-8/SVneo cell migration and invasion after 48 h. **F** Flow cytometry was performed to evaluate transfected HTR-8/SVneo cell apoptosis after 48 h. Data presented as mean ± SD. *p < 0.05, **p < 0.01, ***p < 0.001

To verify the expression of tRF-5028c in HTR-8/SVneo cells, FISH was first performed, and it was observed that tRF-5028c was located in both cytoplasm and nucleus (Fig. 2A). To investigate the role of tRF-5028c in regulating trophoblast function, tRF-5028c knockdown/overexpression was then induced by transfecting its inhibitor/mimic into HTR-8/SVneo cells. Compared with NCs, the inhibitor and mimic of tRF-5028c were found to significantly reduce and elevate its expression after 24 h (Fig. 2B, C), indicating the transfection was successful. Functional experiments subsequently showed that tRF-5028c knockdown markedly promoted trophoblast proliferation, migration, and invasion (Fig. 2D, E). Moreover, cell apoptosis was detected by Annexin V-FITC/PI assay after 48 h of transfection, and the results revealed that tRF-5028c knockdown inhibited apoptosis, while tRF-5028c overexpression displayed the opposite effects (Fig. 2F). Collectively, these results suggested that tRF-5028c upregulation impaired trophoblast cell function in RSA.

### tRF-5028c suppressed CRKL expression by directly targeting CRKL

**Fig. 3:**
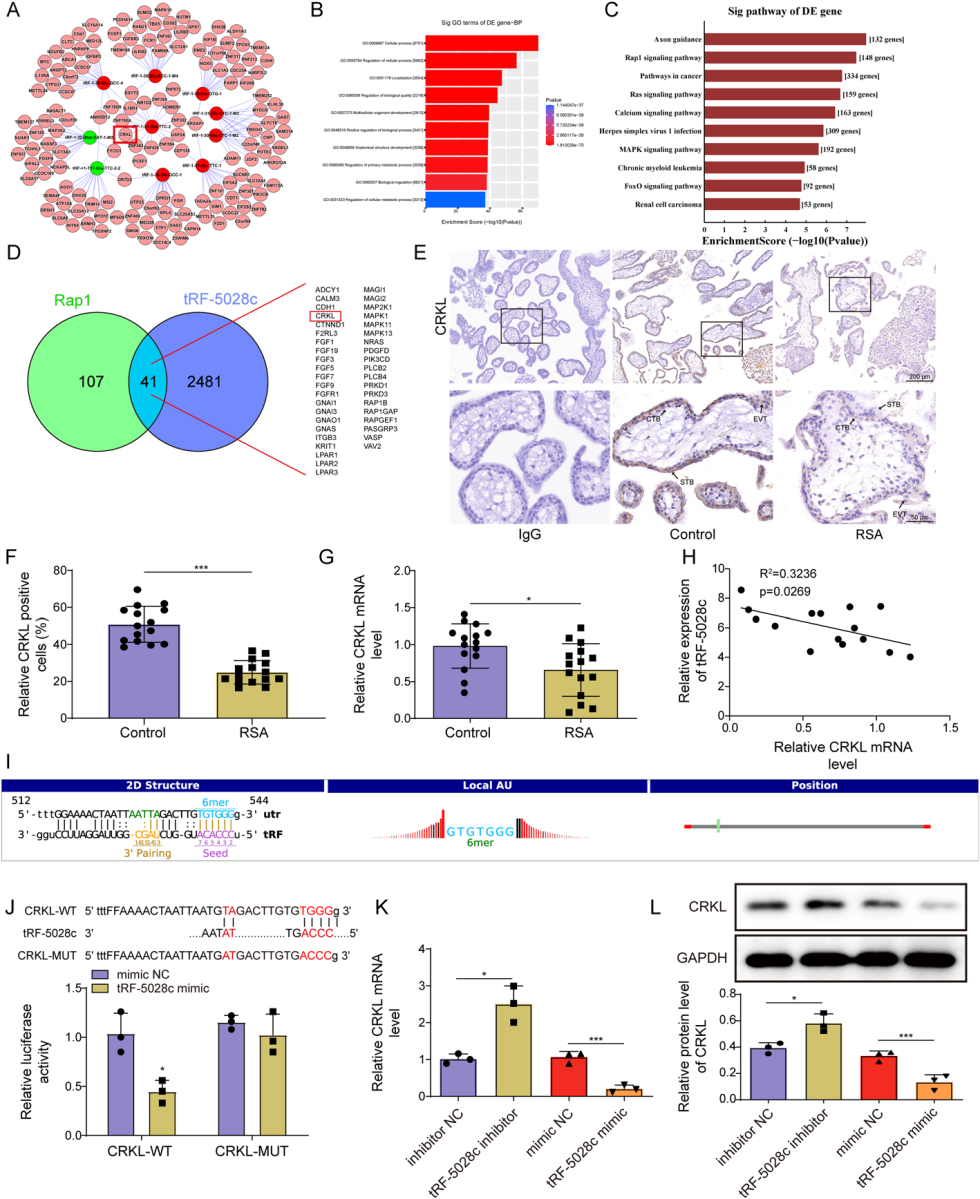
tRF-5028c suppressed CRKL expression by directly targeting CRKL. **A** Prediction of tsRNA target genes and network construction based on miRanda and TargetScan. The red dots represent the eight upregulated tsRNAs, the green dots represent the two downregulated tsRNAs, and the pink dots represent their predicted target genes. **B** GO analysis of biological processes of tsRNA target genes. **C** KEGG analysis of signaling pathways of tsRNA target genes. **D** Venn diagram showing the intersection of tRF-5028c target genes with the Rap1 pathway genes. **E** Representative IHC images of villous expression and localization of CRKL in patients with RSA (*n* = 15) and healthy controls (*n* = 15). IgG is used as an isotype control, and the arrows indicate different types of trophoblasts (CTB, STB, and EVT). **F** Quantification of the percentage of CRKL-positive cells based on IHC staining. **G** The mRNA levels of CRKL in the villous tissues of patients with RSA (*n* = 15) and healthy controls (*n* = 15) were detected by qRT-PCR. **H** Pearson correlation analysis of tRF-5028c and CRKL mRNA levels in the villous tissues of patients with RSA (*n* = 15). **I** A 2D map of the potential binding sites between tRF-5028c and CRKL was obtained by integrating the algorithms of miRanda and TargetScan. The 5′ seed sequence of tRF-5028c (position 2–7, purple) is complementary to the 3′-UTR of CRKL mRNA (TGTGGG, blue). **J** The 3′-UTR of CRKL and the mutated sequence (shown in red) were cloned into the pGL3 vector to construct wild-type (WT) and mutant (MUT) reporter plasmids. Dual luciferase reporter gene assay was performed to analyze the interaction between tRF-5028c and CRKL. **K**, **L** HTR-8/SVneo cells were transfected with tRF-5028c inhibitor/mimic or inhibitor/mimic NC for 48 h, and the mRNA and protein levels of CRKL were detected by qRT-PCR and western blot, respectively. Data presented as mean ± SD. *p < 0.05, ***p < 0.001

The downstream targets of tRF-5028c in trophoblast biology were then explored. First, we used miRanda and TargetScan databases to obtain the target genes of ten tsRNAs screened above (Supplementary Table S3), and the tsRNA-mRNA network was briefly presented in Fig. 3A. Further GO and KEGG analyses showed that these genes (*n* = 10,435) were mainly enriched in the regulation of cellular and biological processes as well as diverse signaling pathways, especially Rap1 (Fig. 3B, C). Given the established role of Rap1 activation in trophoblast function [17], we subsequently intersected the target genes of tRF-5028c with the Rap1 pathway genes. A total of 41 candidate genes were obtained (Fig. 3D). Among them, CRKL is directly functional for Rap1 activation [21], widely expressed in human tissues including placenta [22] and actively involved in regulating tumor cell behavior similar to EVT [23]. However, no studies have reported the association between CRKL and RSA. Therefore, CRKL was selected as the potential target gene of tRF-5028c for further exploration.

As shown in Fig. 3E–G, both IHC and qRT-PCR analyses demonstrated that CRKL expression levels were markedly decreased in the first-trimester villous tissues of patients with RSA (*n* = 15) compared with controls (*n* = 15). IHC also indicated that CRKL was expressed in CTB, STB, and EVT (Fig. 3E). To further determine its localization, we performed double immunofluorescence staining with CTB marker CK7 or EVT marker HLA-G, both of which displayed co-localization with CRKL on human villous tissues (Supplementary Fig. S1). In addition, a significantly negative correlation was observed between CRKL mRNA and tRF-5028c expressions among patients with RSA (Fig. 3H).

Similar to miRNAs, the activity of tsRNAs is largely determined by the complementary pairing between their 5′ seed sequence (position 2–7) and the 3′-UTR of the target mRNA [24]. tRF-5028c was predicted to have potential binding sites to CRKL (TGTGGG) (Fig. 3I). As confirmed by dual luciferase reporter gene assay, tRF-5028c overexpression significantly decreased the luciferase activity of CRKL-WT but did not affect that of CRKL-MUT (Fig. 3J). Consistently, tRF-5028c knockdown markedly increased CRKL expression in trophoblast cells, whereas its overexpression reduced CRKL levels (Fig. 3K, L). Taken together, tRF-5028c could directly target CRKL 3′-UTR region and suppress its expression.

### tRF-5028c modulated trophoblast function by inhibiting CRKL expression

**Fig. 4:**
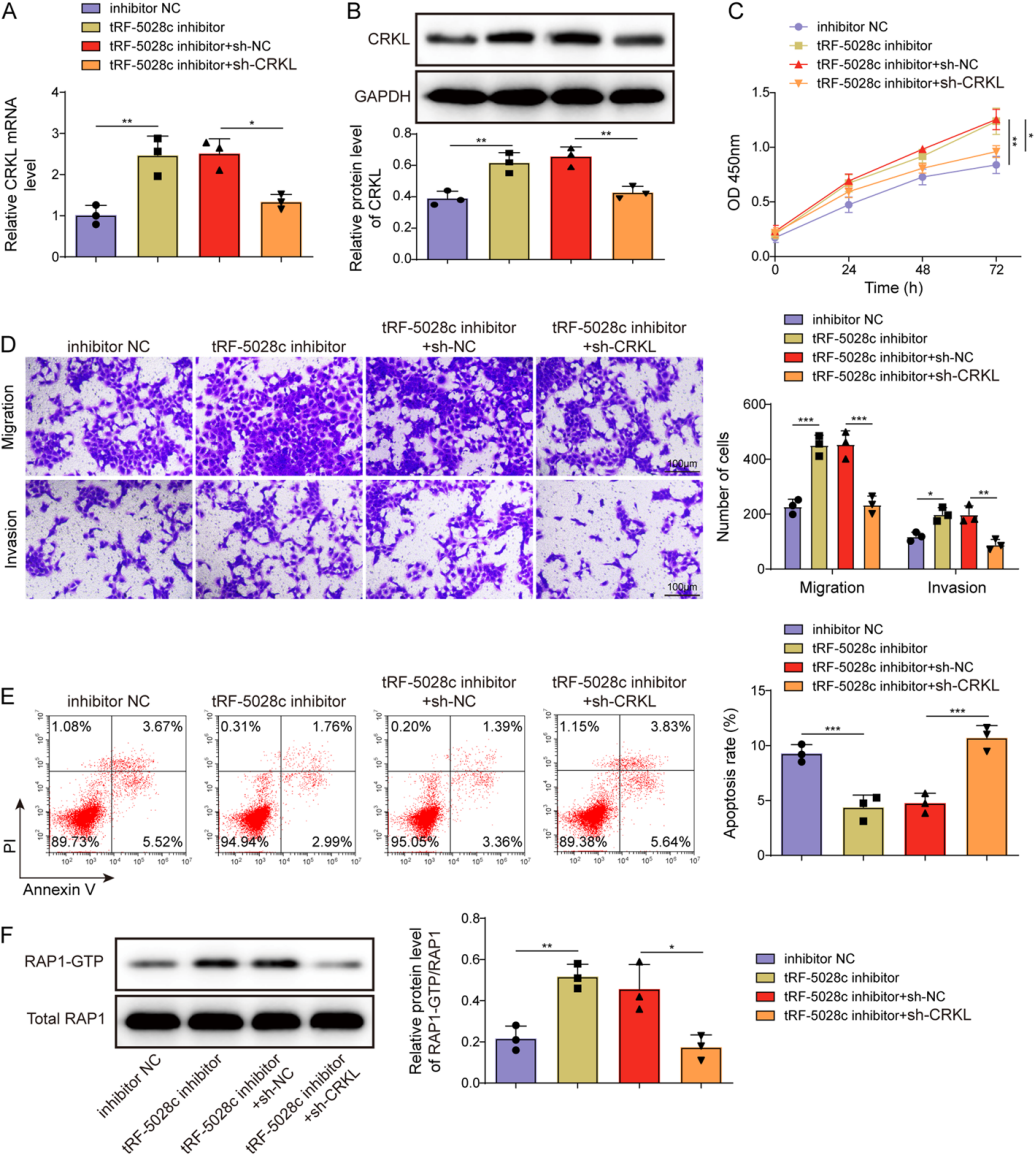
tRF-5028c regulated trophoblast proliferation, migration, invasion, and apoptosis by inhibiting CRKL expression. HTR-8/SVneo cells were co-transfected with inhibitor NC or tRF-5028c inhibitor and sh-NC or sh-CRKL. **A**, **B** The mRNA and protein levels of CRKL in transfected HTR-8/SVneo cells were assessed by qRT-PCR and western blot after 48 h. **C** CCK-8 assay was performed to evaluate transfected HTR-8/SVneo cell proliferation at 0, 24, 48, and 72 h. **D** Transwell assay was performed to evaluate transfected HTR-8/SVneo cell migration and invasion after 48 h. **E** Flow cytometry was performed to evaluate transfected HTR-8/SVneo cell apoptosis after 48 h. **F** Rap1-GTP activity was detected using pull-down assay in transfected HTR-8/SVneo cell after 48 h. Data presented as mean ± SD. *p < 0.05, **p < 0.01, ***p < 0.001

To study the interaction between tRF-5028c and CRKL in trophoblast function, both tRF-5028c and CRKL knockdown were induced by transfecting tRF-5028c inhibitor and sh-CRKL into HTR-8/SVneo cells. After 48 h, CRKL expression level in transfected cells was detected by qRT-PCR and western blot, and the results showed that tRF-5028c inhibitor markedly increased CRKL expression, while this change was eliminated by sh-CRKL co-transfection (Fig. 4A, B). CRKL knockdown rescued the phenotypes of tRF-5028c silencing on trophoblast proliferation, migration, and invasion (Fig. 4C, D). Moreover, the inhibitory effect of tRF-5028c knockdown on trophoblast apoptosis was reversed by CRKL knockdown (Fig. 4E). tRF-5028c inhibitor was also shown to increase the Rap1-GTP activity in trophoblast, while sh-CRKL co-transfection reversed the promoting effect (Fig. 4F). In summary, tRF-5028c modulated trophoblast function via downregulating expression of CRKL.

### CRKL governed trophoblast function by activating the C3G/Rap1 pathway

**Fig. 5:**
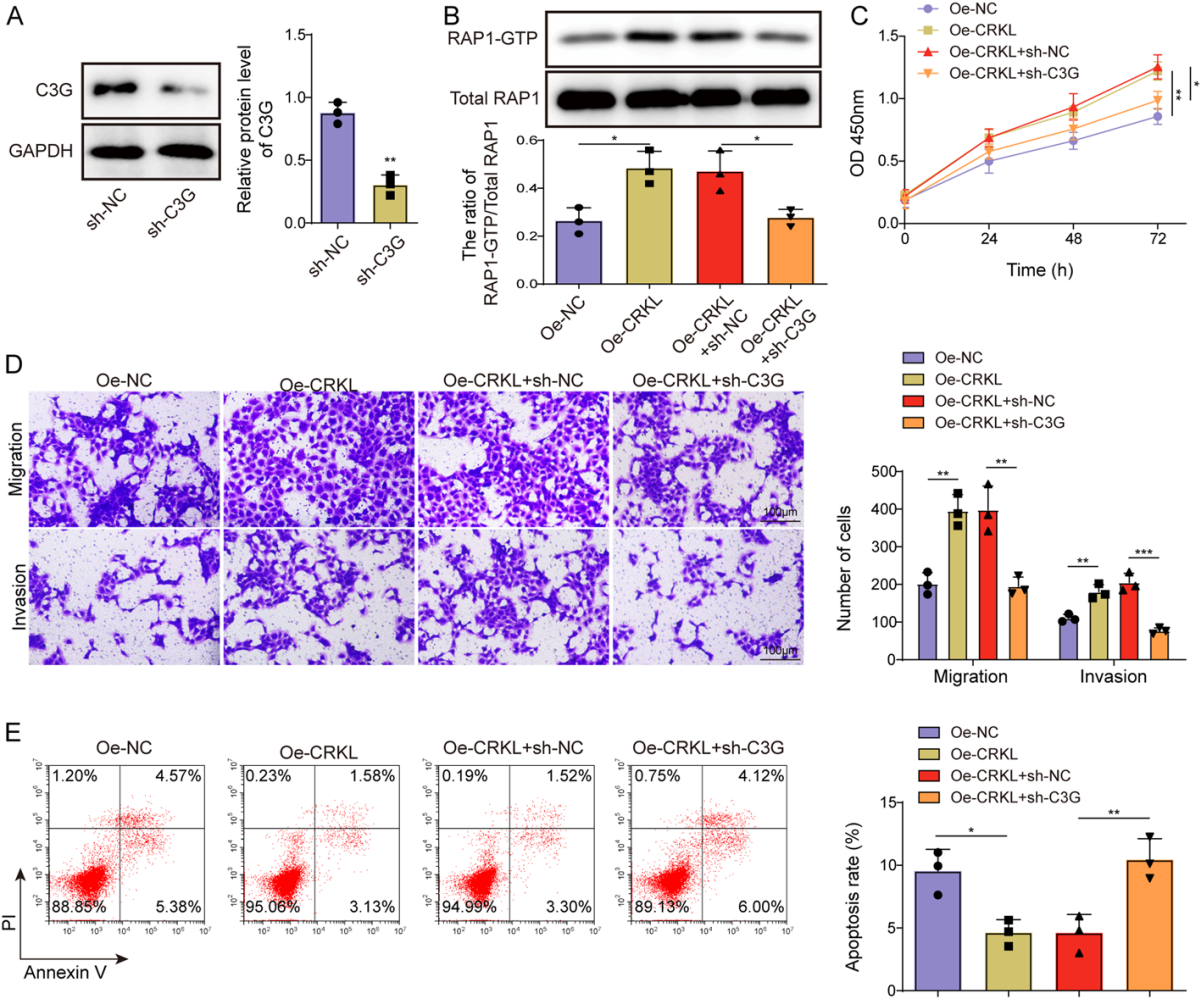
CRKL regulated trophoblast proliferation, migration, invasion, and apoptosis by activating the C3G/Rap1 signaling pathway. **A** HTR-8/SVneo cells were transfected with sh-C3G or sh-NC, and the knockdown efficiency of C3G was verified by Western blot after 48 h. HTR-8/SVneo cells were co-transfected with Oe-NC or Oe-CRKL and sh-NC or sh-C3G. **B** Rap1-GTP activity was detected using pull-down assay after 48 h of transfection. **C** CCK-8 assay was performed to evaluate transfected HTR-8/SVneo cell proliferation at 0, 24, 48, and 72 h. **D** Transwell assay was performed to evaluate transfected HTR-8/SVneo cell migration and invasion after 48 h. **E** Flow cytometry was performed to evaluate transfected HTR-8/SVneo cell apoptosis after 48 h. Data presented as mean ± SD. *p < 0.05, **p < 0.01, ***p < 0.001

Rap guanine nucleotide exchange factor 1 (RAPGEF1), also known as C3G, is recognized as a Rap1 activator by promoting the exchange of GDP for GTP. As an adaptor protein, CRKL can interact with the proline-rich region of C3G through its SH3 domain, thus localizing C3G to specific areas of the cell for activation [25]. Herein, we investigated the involvement of CRKL/C3G/Rap1 axis in regulating trophoblast cell function. The efficiency of sh-C3G transfection is shown in Fig. 5A. HTR-8/SVneo cells were then co-transfected with sh-C3G and oe-CRKL for 48 h. Rap1-GTP activity in trophoblasts was markedly elevated by CRKL overexpression, but was abolished following C3G silencing (Fig. 5B). As evidenced by CCK-8, transwell, and flow cytometry analyses, C3G knockdown also ameliorated oe-CRKL-induced increase in trophoblast proliferation, migration, and invasion while reversing the decrease in cell apoptosis (Fig. 5C–E). Therefore, CRKL enhanced trophoblast cell function by activating the C3G/Rap1 signaling pathway.

### tRF-5028c overexpression increased embryo absorption in mice

**Fig. 6:**
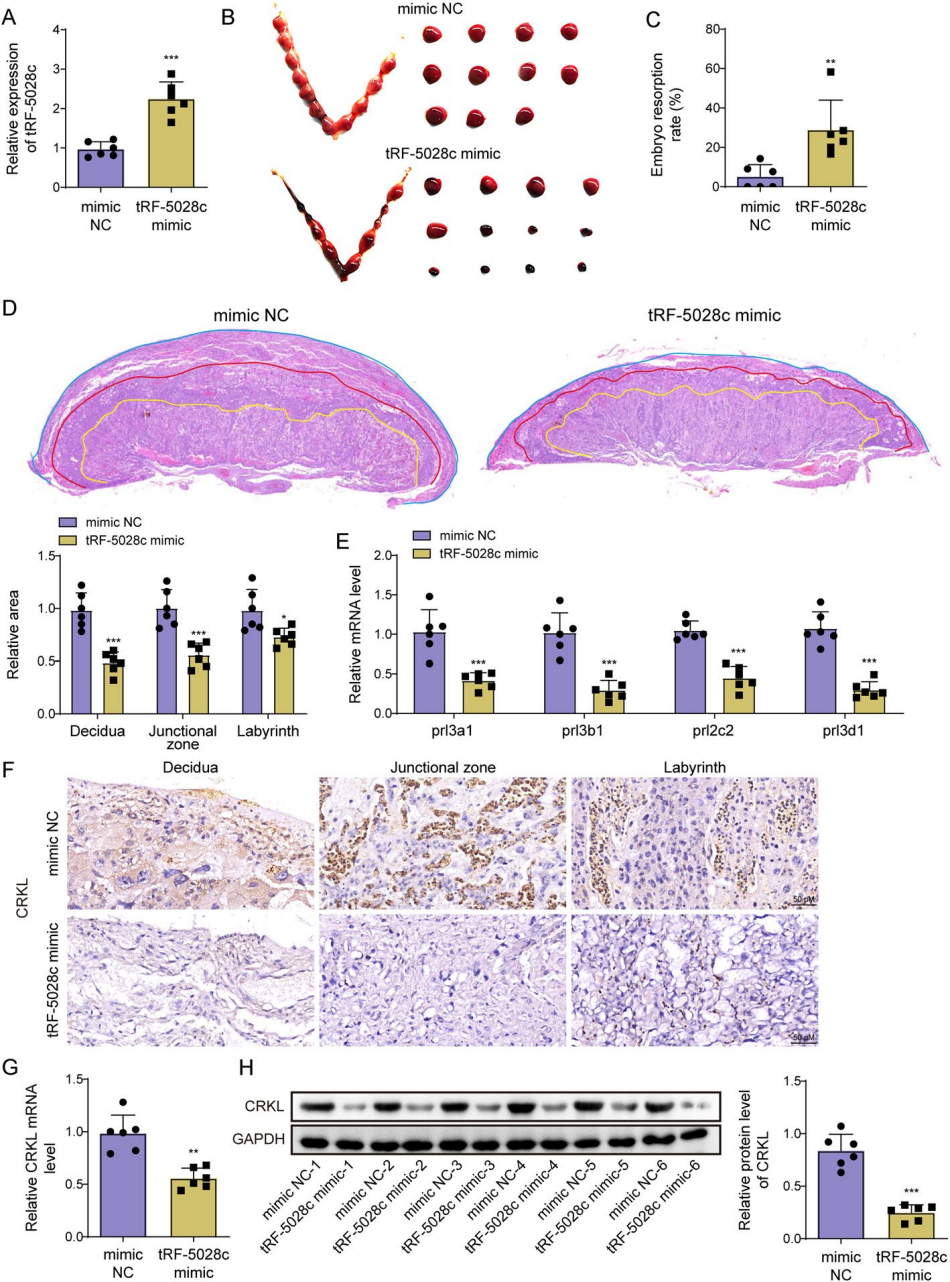
tRF-5028c overexpression increased embryo absorption in mice. Pregnant mice were injected with 10 nmol of tRF-5028c mimic (*n* = 6) or mimic NC (*n* = 6) on GD5.5, GD8.5, and GD11.5 via tail vein, and euthanized on GD13.5. **A** Placental tRF-5028c level was detected by qRT-PCR. **B**, **C** Analysis of embryo adsorption rate on GD13.5. **D** Representative images of HE staining in the placentas. The decidua, junctional zone, and labyrinth layers are marked with blue, red and yellow lines, and measured for areas, respectively. **E** The mRNA levels of invasion-related marker genes (*prl3a1*, *prl3b1*, *prl2c2*, and *prl3d1*) in placental tissues were assessed using qRT-PCR. **F** Representative IHC images of expression and localization of CRKL in mice placentas. **G**, **H** Placental mRNA and protein levels of CRKL were detected by qRT-PCR and Western blot, respectively. Data presented as mean ± SD. *p < 0.05, **p < 0.01, ***p < 0.001

To further confirm the in vitro findings, pregnant mice were injected with 10 nmol of tRF-5028c mimic or mimic NC on GD5.5, GD8.5, and GD11.5 via tail vein. On GD13.5, mice were euthanized to assess the embryo absorption rate and the placental tissues were collected for further analysis. As displayed in Fig. 6A, tRF-5028c was indeed overexpressed in mice injected with tRF-5028c mimic. Moreover, tRF-5028c significantly increased the embryo adsorption rates compared with NC (Fig. 6B, C). Placental histological analysis demonstrated that the areas of decidua, junctional zone, and labyrinth were all significantly reduced after tRF-5028c overexpression (Fig. 6D). In addition, the results of qRT-PCR revealed a remarkable downregulation of invasion-related marker genes, including *prl3a1*, *prl3b1*, *prl2c2*, and *prl3d1* (Fig. 6E). IHC staining further showed that CRKL could be detected in trophoblasts and other cell types within the three functional layers (Fig. 6F). Moreover, in comparison with the mimic NC group, the tRF-5028c mimic group reduced CRKL mRNA and protein levels in mice placentas (Fig. 6G, H). In summary, tRF-5028c upregulation promoted embryo absorption in vivo.

## Discussion

RSA is a distressing disorder that affects around 1–5% of women attempting to conceive and has a significant impact on quality of life [2, 26]. Trophoblast cells play key biological roles in placental anchoring, maternal spiral artery modeling, as well as hormone secretion in early pregnancy [27]. Dysfunction of trophoblasts has been implied in the pathogenesis of RSA and suggested as a potential treatment target [28]. Through a combination of bioinformatic, molecular, cellular, and animal evidence, the present study shows that tRF-5028c upregulation disrupted trophoblast biology in RSA by inhibiting CRKL-mediated Rap1 signaling pathway.

tsRNAs present a novel class of noncoding RNA family and are actively functional in various biological processes [29]. Dysregulated tsRNAs have been observed in the progression of multiple diseases [9–13], while the role of tsRNAs in RSA has not been reported. In this study, high-throughput sequencing was performed in the villous tissues of patients with RSA and healthy controls, which revealed significantly differential tsRNA expression profiles. Among them, tRF-5028c knockdown in vitro enhanced EVT proliferation, migration, and invasion and inhibited apoptosis. Conversely, its overexpression promoted embryo absorption and suppressed placental development in vivo. All these results indicated that tRF-5028c upregulation in RSA impaired the normal function of trophoblast cells. Given the increasing interest in tsRNAs as predictive biomarkers and therapeutic targets [30], tRF-5028c might serve as a novel candidate for early RSA diagnosis or intervention. While challenges such as RNA delivery and off-target effects need to be addressed, our findings provide a theoretical foundation for further preclinical investigation to explore its feasibility, safety, and efficacy.

Mechanically, tsRNAs could exert RNA silencing effect by inhibiting gene posttranscription via an AGO-RISC approach in a miRNA-like manner [10]. However, rather than AGO2, they preferentially bind to AGO1, AGO3, and AGO4 to enable their interaction with target mRNAs [24]. They also exhibit distinct regulatory features compared to miRNAs, such as specific biogenesis pathways [30]. In this study, we predicted the downstream target of tRF-5028c with miRanda and TargetScan databases based on complementary seed sequences. By dual luciferase reporter assay, we demonstrated that tRF-5028c could bind with the 3′-UTR region of CRKL mRNA to suppress its expression. Therefore, CRKL was identified as a direct target gene of tRF-5028c.

CRKL is an adaptor protein widely expressed in human tissues, but its function in trophoblast and placental biology remains unclear. Previous studies have revealed CRKL’s involvement in key signaling pathways, offering insights into its potential roles in pregnancy-related processes. For instance, CRKL was identified as a transcriptional target of Hh-GLI2 pathway in lung adenocarcinoma and played an essential role in GLI2-driven cell proliferation and migration [31]. CRKL also promoted hepatocarcinoma via PI3K/Akt-mediated glucose metabolism, including glucose uptake, lactate production, and glycogen synthesis [32]. Analogously, it has been found that CRKL regulated neutrophil adhesion, brain neuron development, T cell receptor signaling, as well as tumorigenesis of head and neck squamous cell carcinoma via binding to C3G and activating the Rap1 pathway [33–36]. These findings highlight CRKL’s capability to modulate cellular behavior, metabolic function, as well as immune tolerance, all of which are critical for pregnancy establishment and maintenance. Here, we demonstrated that the CRKL/C3G axis could also promote EVT proliferation, migration, and invasion while inhibiting apoptosis. Moreover, CRKL downregulation in RSA villous tissues aligns with its potential role in poor placentation. The effect was further related to the increased Rap1 activity, which was abolished by tRF-5028c. As a downstream effector of the CRKL/C3G axis, Rap1 has been implicated in pregnancy complications such as preeclampsia and gestational diabetes mellitus [37, 38], yet direct studies on RSA remain to be reported. Thus, our findings underscore a possible mechanistic link between tRF-5028c-mediated suppression of CRKL/Rap1 signaling and the impaired trophoblast function observed in RSA.

Despite these insights, certain limitations should be highlighted for attention. Firstly, the timing of sampling differed between two groups, where the villous tissues were obtained after abortion from patients with RSA but before abortion (viable) from healthy controls. This difference could result in a possible confounding effect on tsRNA expression, and further studies should address this critical issue with ethical considerations. Secondly, the linear tRF-5028c-CRKL “axis” may oversimplify the complex regulatory network involved. Beyond this direct interaction, it is important to consider the broader involvement of a functionally integrated competitive endogenous RNA (ceRNA) interactome, which is ubiquitously present in cells and plays a critical role in defining phenotypes [39]. Future studies should explore how tRF-5028c integrates within this interactome and whether it acts synergistically with other tsRNAs or miRNAs to influence CRKL expression and related pathways. This perspective could provide deeper insight into the molecular mechanisms underlying RSA and identify additional therapeutic targets. Finally, tRNAs undergo various modification patterns that affect their stability, folding, localization, and decoding [40]. As cleaved products, endogenous tsRNAs could also harbor more than one tRNA modifications, such as 5-methylcytosine (m5C), N1-methyladenosine (m1A), N7-methylguanosine (m7G), and pseudouridine (Ψ) [41]. Compared with unmodified tsRNAs, the modified tsRNAs exhibit distinct modification-dependent effects in biological regulation. For instance, TRMT6/61A-dependent m1A modification on tRF-3 was found to restrain RNA silencing, thereby increasing the expression of unfolded protein response genes in bladder cancer [42]. Moreover, METTL1-mediated m7G-3′-tiRNA LysTTT promoted bladder cancer malignancy by binding to ANXA2 to enhance its phosphorylation by Yes1 [43]. However, our study only investigated the differential tsRNAs in RSA, while their modifications remain unclear and warrant further exploration with specialized secondary sequencing techniques [41]. In conclusion, our research showed that tRF-5028c upregulation in RSA impaired trophoblast function by directly targeting CRKL and suppressing C3G/Rap1 signaling pathway. The findings expand the mechanism of noncoding RNAs in RSA pathogenesis, and could hopefully provide new targets for its diagnosis and treatment.

## Supplementary Information

Supplementary Material 1: Table S1. Characteristics of the control and patients with RSA. Table S2. The primer sequences for qRT-PCR.

Supplementary Material 2: Table S3. Prediction of target genes of 10 tsRNA candidates based on miRanda and TargetScan.

Supplementary Material 3: Figure S1. Representative images of double immunofluorescence staining of CRKL (green) and CK7 or HLA-G (red) in first-trimester villous tissues.

## Data Availability

The data analyzed in this study can be obtained from authors by email (huangjialv_medicine@foxmail.com) upon reasonable request.

## Abbreviations

CTB: Cytotrophoblast
STB: Syncytiotrophoblast
AGO: Argonaute
RSA: Recurrent spontaneous abortion
tsRNAs: TRNA-derived small RNAs
tiRNAs: TRNA halves
tRFs: TRNA-derived fragments
Rap1: Ras‐associated protein‐1
CRKL: v-crk sarcoma virus CT10 oncogene homologue-linke
IHC: Immunohistochemistry
FISH: Fluorescence in situ hybridization
CCK-8: Cell counting kit-8
qRT-PCR: Quantitative real-time polymerase chain reaction
EVT: Extravillous trophoblast
CPM: Counts per million mapped reads
GO: Gene Ontology
KEGG: Kyoto Encyclopedia of Genes and Genomes
NC: Negative control
WT: Wild type
Mut: Mutant
RISC: RNA-induced silencing complex
