# Supplementary material for: tRF-5028c disrupts trophoblast function in recurrent spontaneous abortion by inhibiting CRKL-mediated Rap1 signaling pathway
Source: Cell Mol Biol Lett. 2025 Mar 5;30:28. doi: 10.1186/s11658-025-00706-w (PMC11881442; doi:10.1186/s11658-025-00706-w)
Supplement: Supplementary file 1 — Supplementary Material 1: Table S1. Characteristics of the control and patients with RSA. Table S2. The primer sequences for qRT-PCR. [file 11658_2025_706_MOESM1_ESM.docx]

**Table S****1. Characteristics of the control and RSA patients.**

| **Characteristics** | **Control (*n* = 15)** | **RSA (*n* = 15)** | ***P*-value** |
| --- | --- | --- | --- |
| Age (years) | 30.6±4.3 | 31.2±4.3 | 0.706 |
| Body mass index (kg/m^2^) | 21.1±1.5 | 22.0±1.6 | 0.121 |
| Gestational age (days) | 59.3±8.1 | 56.1±8.2 | 0.283 |
| Gravidity | 2.1±0.7 | 3.7±0.8 | <0.001 |
| Spontaneous abortion | 0 | 3.2±0.7 | NA |
| Parity | 2.1±0.7 | 0.5±0.7 | <0.001 |

Data were presented as mean ± SD.

Abbreviations: RSA, recurrent spontaneous abortion; NA, not applicable.

**Table S2. The primer sequences for qRT-PCR.**

| **Genes** | | **Sequence (5’-3’)** |
| --- | --- | --- |
| tRF-5028c-RT | GTCGTATCCAGTGCAGGGTCCGAGGTATTCGCACTG | |
| tRF-5028c-F | GCCGAGTCCCACATGGTCTAGCGGTTAGGAT | |
| tRF-1:30-Glu-CTC-1-M2-RT | GTCGTATCCAGTGCAGGGTCCGAGGTATTCGCACTGGATACGACCGCCGA | |
| tRF-1:30-Glu-CTC-1-M2-F | GCCGAGTCCCTGGTGGTCTAGTGGTTAGGAT | |
| tRF-1:30-Gly-GCC-1-RT | GTCGTATCCAGTGCAGGGTCCGAGGTATTCGCACTGGATACGACGCGAGA | |
| tRF-1:30-Gly-GCC-1-F | GCCGAGGCATGGGTGGTTCAGTGGTAGAAT | |
| tRF-1:31-Glu-TTC-1-RT | GTCGTATCCAGTGCAGGGTCCGAGGTATTCGCACTGGATACGACCCAGGA | |
| tRF-1:31-Glu-TTC-1-F | GCCGAGTCCCATATGGTCTAGCGGTTAGGAT | |
| tRF-1:30-Gly-GCC-4-RT | GTCGTATCCAGTGCAGGGTCCGAGGTATTCGCACTGGATACGACGCAAGA | |
| tRF-1:30-Gly-GCC-4-F | GCCGAGGCATAGGTGGTTCAGTGGTAGAAT | |
| tRF-1:30-Gly-CCC-1-M4-RT | GTCGTATCCAGTGCAGGGTCCGAGGTATTCGCACTGGATACGACGCGAGA | |
| tRF-1:30-Gly-CCC-1-M4-F | GCCGAGGCATTGGTGGTTCAGTGGTAGAAT | |
| tRF-1:32-iMet-CAT-1-M2-RT | GTCGTATCCAGTGCAGGGTCCGAGGTATTCGCACTGGATACGACGGCCCA | |
| tRF-1:32-iMet-CAT-1-M2-F | GCCGAGAGCAGAGTGGCGCAGCGGAAGCGTGC | |
| tRNA -R | GTGCAGGGTCCGAGGT | |
| U6-R | AACGCTTCACGAATTTGCGT | |
| U6-F | CTCGCTTCGGCAGCACA | |
| Human-CRKL-R | GGTTGGGTGCTGAGACAGAT | |
| Human-CRKL-F | CGGGTCTCCCACTACATCAT | |
| Human-GAPDH-R | TGACGGTGCCATGGAATTTG | |
| Human-GAPDH-F | AGGTCGGAGTCAACGGATTT | |
| Mouse-CRKL-R | CCGGACTAGGAACATGCCAT | |
| Mouse-CRKL-F | TCCTCCGCCAGGTTTGATTC | |
| Mouse-prl3a1-R | CATCTGCCAGTCCCATCCAA | |
| Mouse-prl3a1-F | GAAGGAGCCTGCAAGACCAT | |
| Mouse-prl3b1-R | AGGTACATGTGGAAGAGCAGC | |
| Mouse-prl3b1-F | CCAGAAAACAGCGAGCAAGT | |
| Mouse-prl2c2-R | TCTCATGGGGCTTTTGTCTC | |
| Mouse-prl2c2-F | TGAGGAATGGTCGTTGCTTT | |
| Mouse-prl3d1-R | CAGGGGAAGTGTTCTGTCTGT | |
| Mouse-prl3d1-F | TGGTGTCAAGCCTACTCCTTT | |
| Mouse-gapdh-R | AAGAGTGGGAGTTGCTGTTGAAG | |
| Mouse-gapdh-F | GGAGAAACCTGCCAAGTATGATG | |
