# Supplementary figures and images for: tRF-5028c disrupts trophoblast function in recurrent spontaneous abortion by inhibiting CRKL-mediated Rap1 signaling pathway
Source: Cell Mol Biol Lett. 2025 Mar 5;30:28. doi: 10.1186/s11658-025-00706-w (PMC11881442; doi:10.1186/s11658-025-00706-w)

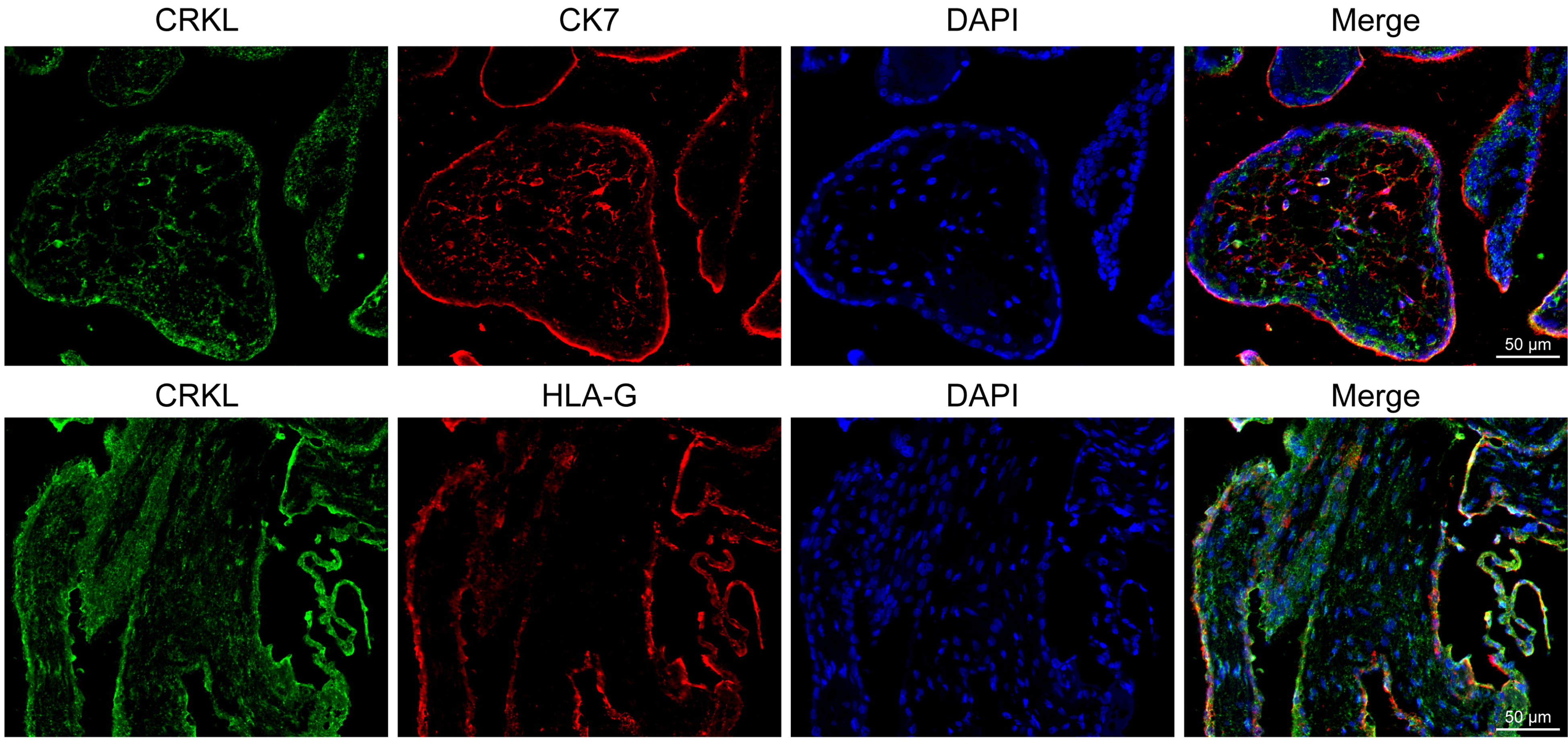

Supplement: Supplementary file 3 — Supplementary Material 3: Figure S1. Representative images of double immunofluorescence staining of CRKL (green) and CK7 or HLA-G (red) in first-trimester villous tissues. [file 11658_2025_706_MOESM3_ESM.tif]
